# Hepatitis C virus seropositivity and genotyping by RealTime PCR in a tertiary care hospital in Southern India

**DOI:** 10.1186/1471-2334-14-S3-P39

**Published:** 2014-05-27

**Authors:** SR Ramya, M Kulkarni, GS Vijay Kumar

**Affiliations:** 1 Department of Microbiology, JSS Medical College, Mysore, Karnataka, India

## Background

Hepatitis C virus is one of the major causes of parenterally acquired hepatitis. HCV genotypes exhibit different profiles of pathogenicity, infectivity and response to antiviral therapy. The study was taken up to detect HCV seropositivity and common genotype prevalence in a tertiary care hospital. The other objectives of the study were to detect probable risk factors and HIV/HBV co infections.

## Methods

The duration of study was 2 years. Adult patients clinically suspected of hepatitis, blood donors attending blood bank, pregnant women attending ANC clinic, renal dialysis patients and patients whose blood samples were received for pre-operative evaluation, were the subjects of the study. Serum was subjected to ELISA for anti-HCV antibody detection. Plasma collected from seropositives, genotyped by RealTime PCR. In accordance with prevalence of seropositivity same number of controls were randomly selected and analysis done for association with risk factors using Chi square test.

## Results

Of the 3581 serum samples screened, 32 were anti-HCV antibody positive (0.89%). Highest number of HCV seropositivity found between 41-50 years (40.62%). Blood transfusion was found to be highly significant risk factor (*p* value 0.004). Rate of HCV-HBV co infection was 6.25% (2/32) and HCV-HIV co infection was 3.12% (1/32). No concomitant HCV, HIV and HBV infection was detected. 12 samples were HCV Genotype 1(60%), 8 were Genotype 3 (40%) and 5 were untypeable.

## Conclusion

Blood transfusion is a major risk factor for HCV infection and genotype 1 predominates over genotype 3 in this region.

